# Implicit and Explicit Learning of a Sequential Postural Weight-Shifting Task in Young and Older Adults

**DOI:** 10.3389/fpsyg.2016.00733

**Published:** 2016-05-24

**Authors:** Simone R. Caljouw, Renee Veldkamp, Claudine J. C. Lamoth

**Affiliations:** Center for Human Movement Sciences, University Medical Center Groningen, University of Groningen, GroningenNetherlands

**Keywords:** implicit motor learning, postural control, aging, older adults, sequence learning

## Abstract

Sequence-specific postural motor learning in a target-directed weight-shifting task in 12 older and 12 young participants was assessed. In the implicit sequence learning condition participants performed a concurrent spatial cognitive task and in the two explicit conditions participants were required to discover the sequence order either with or without the concurrent cognitive task. Participants moved a cursor on the screen from the center location to one of the target locations projected in a semi-circle and back by shifting their center of pressure (CoP) on force plates. During the training the targets appeared in a simple fixed 5-target sequence. Plan-based control (i.e., direction of the CoP displacement in the first part of the target-directed movement) improved by anticipating the sequence order in the implicit condition but not in the explicit dual task condition. Only the young participants were able to use the explicit knowledge of the sequence structure to improve the directional error as indicated by a significant decrease in directional error over practice and an increase in directional error with sequence removal in the explicit single task condition. Time spent in the second part of the movement trajectory to stabilize the cursor on the target location improved over training in both the implicit and explicit sequence learning conditions, for both age groups. These results might indicate that an implicit motor learning method, which holds back explicit awareness of task relevant features, may be desirable for improving plan-based motor control in older adults.

## Introduction

Repeating patterns or sequences occur often in our environments, and in many activities of daily living a sequence of individual acts is performed in interaction with the environment. For example, the sequence of asks needed to get a glass of water may include, leaning over to a cupboard, opening the cupboard, grasping a glass, moving to the tap, and turning on the faucet. Prediction of, adaptation to, and learning about, environmental regularities on the basis of preceding events may assist in how the body needs to be moved in order to achieve the task goal, and requires adequate postural control (Sturnieks et al., 2008; de Vries et al., 2014).

Aging yields an undeniable deterioration of postural control, as a consequence of a general age-related deterioration of sensory and neuromuscular control mechanisms (Laughton et al., 2003; Sturnieks et al., 2008). Consequently, maintaining postural stability during daily tasks becomes less automatic, and requires increased attention. Aging, however, is not only associated with a decline in postural control, but also with a deterioration of cognitive processes involving executive functions and attention (Castel and Craik, 2003; Park et al., 2003; Lovden et al., 2008; Verwey, 2010). Thus, older adults have greater need for conscious attention to maintain good postural control, due to impaired sensory and motor system functions. At the same time, they suffer from reduced attentional and working memory capacity. Since postural control becomes less automatic, depending on the complexity of the motor task, the execution of a concurrent cognitive task will lead to performance decrements (Huxhold et al., 2006; Boisgontier et al., 2013).

The age-related decline of cognitive functions makes it plausible that the ability to explicitly learn sequential motor skills decreases with age, since explicit motor learning requires an intention to learn and thus a contribution of strategic processes such as attention, reasoning, and memory (Fitts and Posner, 1967). Young adults acquire more explicit knowledge about the sequence structure than older adults (Howard and Howard, 2001; Verneau et al., 2014). When older adults do acquire explicit knowledge about a sequence, they would be less successful in using this knowledge (Shea et al., 2006; Verneau et al., 2014). Implicit learning, on the other hand, is considered to depend on a phylogenetically older and more primitive system than explicit learning (Reber, 1992). In implicit learning, learning occurs without an intention to learn and without explicit knowledge about the environmental regularities, it thus depends less on the working memory capacity (Masters, 1992; Jimenez and Vazquez, 2005; Janacsek and Nemeth, 2012). Therefore, it is suggested that implicit learning is more robust to the effects of age (Cherry and Stadler, 1995; Song et al., 2009). Abundant research exist on the influence of age on implicit motor learning used serial reaction time tasks (Nissen and Bullemer, 1987; Willingham and Goedert-Eschmann, 1999). In serial reaction time tasks participants have to react as fast as possible on certain stimuli by pressing keys on a keyboard, repeating sequences of stimuli are hidden between random stimuli, in order to remain unknown to the participants (for reviews see: Rieckmann and Backman, 2009; Howard and Howard, 2013; King et al., 2013). With practice, participants become faster due to general skill learning and sequence specific learning. Sequence-specific learning is indicated by an abrupt increase in response times when the sequential regularity is removed. Older adults do show sequence learning in these tasks, however, the rate and magnitude of learning declines when task conditions become cognitively more demanding. This occurs with increased complexity of the sequence due to alternating random and ordered elements (Curran, 1997; Feeney et al., 2002; Howard et al., 2004; Bennett et al., 2007; Simon et al., 2011) or due to an additional working memory load in the form of a dual-task (Frensch and Miner, 1994; Nejati et al., 2008; King et al., 2013; Vandenbossche et al., 2014).

In contrast to serial reaction time tasks, taking advantage of a repeating sequence of elements to improve postural responses is proven to be difficult. Recent studies (Van Ooteghem et al., 2008, 2010) question earlier positive findings of Shea et al. (2001) who showed segment learning in a visuomotor tracking task in which participants were asked to continuously track a target by controlling their center of pressure (CoP; moving a platform on a stabilometer on which they were standing). Chambaron et al. (2006) suggested that performance improvements in this previous study were not the result of segment-specific visuomotor learning, but could be attributed to methodological flaws, i.e., the selection of a repeating segment that was more easy to perform than the random control segments. The studies of Van Ooteghem et al. (2008, 2010) lend further support for Chambaron et al. (2006) indicating no evidence for sequence specific learning in a postural control task in which participants had to maintain balance in response to a repeating pattern of sequential platform manipulations. On the other hand, Orrell et al. (2006) showed that application of an implicit motor learning technique did improve balance performance on a stabilometer. It is presently not known whether implicit motor learning would occur for postural control tasks in which participants produce postural adjustments to environmental regularities of a sequential nature (instead of reactive to perturbations). Therefore, in the present study, sequence-specific postural motor learning in a target-directed weight-shifting task was assessed, in both older and young participants. Instructing participants to discover a sequence can attenuate the degree to which participants gain awareness of the sequence structure, whereas asking them to concurrently perform a task-irrelevant visuo-spatial memory task can abolish awareness of the sequence structure. Previous studies on motor sequence learning in upper limb tasks indicate that explicit awareness of the sequence order is a prerequisite for sequence-specific movement optimization (Moisello et al., 2009, 2011; Oostwoud Wijdenes et al., 2016). When explicit sequence knowledge is important for sequence-specific motor learning in a postural task one would expect to find interference from an added secondary task, especially in older adults.

In the target-directed weight-shifting task, participants control their CoP on a force platform by shifting their weight in order to move a cursor on a screen in front of them (Jongman et al., 2012; de Vries et al., 2014). Participants are asked to move the cursor to a target that can appear in one of five locations. A specific sequence of targets recurs throughout the practice session. Motor skill in this task requires both adequate planning and execution. One needs to predict the upcoming target location to move efficiently to the right side and great execution skill is required to control the cursor to stabilize on the target location. The directional accuracy of the CoP displacement in the first part of the target-directed movement is a good proxy for the planning accuracy, a higher accuracy of movement direction reflects better anticipation of the target location, demonstrating a greater degree of plan-based motor learning (Ghilardi et al., 2003, 2009). A previous study on target-directed weight-shifting showed that the steadiness of the movement increased (i.e., less velocity peaks and dwell time in the vicinity of the target) when the upcoming target location was predictable compared to when it was not (Jongman et al., 2012). This finding suggests that knowledge of the sequence order and anticipating the target location may improve not only the initial planning of the target-directed movement (e.g., directional accuracy in the first part of the movement), but also the visual guidance or control over the execution of the movement (e.g., homing-time necessary for corrective movements in the vicinity of the target).

In the current study healthy older and young participants repeatedly performed a sequence of voluntary displacements of the CoP to assess whether (1) age affects the capacity for sequence-specific postural motor learning, (2) explicit knowledge about the sequence order leads to better movement optimization than implicit learning, and (3) sequence-specific postural motor learning degrades under dual task conditions. We hypothesized that practicing the sequential target-directed weight shifting task leads to sequence specific improvements in both age groups. Since the attentional cost of postural control increases in older adults, we expected also that beneficial effects of sequence awareness on movement optimization are lower in older individuals, especially when a cognitive task is performed simultaneously.

## Materials and Methods

### Participants

Twelve young adults (23.9 ± 4.3 years) and 12 older adults (67.9 ± 2.5 years) participated in the experiment. Participants had no neurological or orthopedic disorders that might have an effect on cognition or postural control and were able to walk and stand unaided for at least 1 h. The local institution’s ethical committee approved the study and the participants signed informed consent.

### Apparatus and Task Environment

The experiment was conducted in the Computer Assisted Rehabilitation Environment laboratory (CAREN; Motek Medical). Participants stood on two force platforms (Bertec FP4060-08). On a large screen, positioned 2.5 m in front of the participant, a cursor provided online feedback of the CoP displacements of the participant (de Vries et al., 2014). The displacements of the CoP were displayed on the vertical screen as cursor movements from left to right for the medio-lateral component and from top to bottom for the anterior–posterior component. At the start of the experiment participants stood in a natural position with arms at the side and the software positioned the cursor on the center target on the screen goal targets were presented sequentially in one of five possible locations on a hemicycle above the central target (north, east, west, north-east, and north-west). When the cursor touched the displayed target for 200 ms the target disappeared and the next target appeared. The distance between the central target and the radial targets was 72 cm on the screen and the diameter of the target was 18 cm, this corresponded with a CoP-displacement of 0.06 and 0.015 m, respectively. Participants were instructed to move the cursor from the central target to the appearing radial target and move the cursor back to the central target when the radial target disappeared and the central target appeared. They were instructed to make movements as quickly and as accurately as possible. The participants were not allowed to move their feet on the force plate. At the start of the experiment a short training session of 30 radial targets was performed to familiarize the participants with the relationship between their body motions and the cursor displacements.

### Design of the Motor Sequence-Learning Task

**FIGURE 1:**
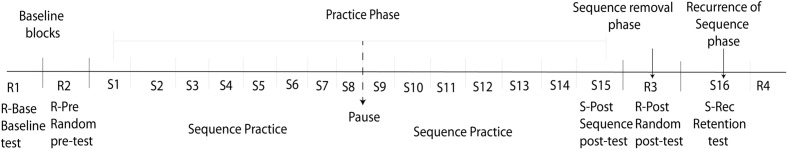
**Description of the experimental setup.** R, random block; S, Sequence block.

In the experimental conditions 20 consecutive blocks were performed in which the targets were presented either in random order (4 R-blocks), or in a fixed recurring sequence (16 S-blocks). See **Figure 1** for an overview of the series of test blocks used in each condition. The targets in the R-block were never presented two times in a row and each target-to-target movement (e.g., neutral to north, neutral to northwest) was presented twice. For each condition the first two test blocks of the experiment were R-blocks consisting of 21 targets. These random blocks were considered baseline blocks; the first random block was the random baseline-test (R-Base) and the second one was used as random pre-test (R-Pre). Subsequently, 15 S-blocks were performed. In each S-block a simple sequence of five different targets was presented three times. Thus, the learning phase consisted of 45 sequence repetitions. The sequence order differed between conditions and participants. The last sequence block (S15) of this learning phase was used as a sequence post-test (S-Post). In-between S-block 8 and S-block 9 a break was introduced to allow for a short-term recovery of fatig. To analyse the effect of sequence removal after sequence learning, S-Post was followed by a R-block of 21 targets (R-Post). After R-Post the sequence was reintroduced in block S16 (S-rec), to reveal if sequence learning was retained after the interfering introduction of a random phase. To diminish effects of sequence expectation, the sequence was reintroduced without any warning and immediately followed by another R-block. (R4). This final R-block was also introduced to prevent participants from obtaining sequence knowledge in retrospect by re-enacting the last trials that were performed.

### The Learning Conditions

Each participant performed this series of blocks three times with different instructions. The order of the conditions was counterbalanced across participants and a rest time of at least 15 min was introduced between learning conditions. In the explicit single-task condition participants were instructed to discover the sequence in the task. In the explicit dual-task condition participants were instructed to discover the sequence in the learning phase and were required to perform a concurrent visuo-spatial memory task. In the implicit condition the participants were instructed that the targets appeared in random order throughout this session and performed a concurrent visuo-spatial memory task. An implicit single-task condition was not included in the current experiment since pilot studies showed that young participants became easily aware of the sequence when the cognitive task was absent.

The visuo-spatial memory task was an adaptation of the Brooks Spatial Matrix task (Brooks, 1967). Participants listened to a set of sentences composing a description of a spatial sequence of locations, such as “In the starting square put a 1, in the next square to the left put a 2, in the next square down put a 3.” For the younger participants, recorded instructions were given for number placement after every 10 target-directed movements, for a total of 17 numbers. Older participants were given instructions after every 13 target-directed movement and were required to remember 13 numbers. This task was administered at the start of the experiment and twice per learning condition (e.g., before and after the break).

After each learning condition it was tested with a free recall test whether the participants acquired awareness of the target sequence. If participants were not able to correctly report the sequence order a four-way forced-choice test was conducted, to test sequence recognition. Prior to the experiment two criteria were formulated regarding the sequence awareness in the different conditions, (1) participants should become aware of the sequence in the explicit single-task condition and (2) participants should not become aware of the sequence in the implicit condition. Participants were excluded from the data analysis when they did not meet these criteria.

### Data Analysis

**FIGURE 2:**
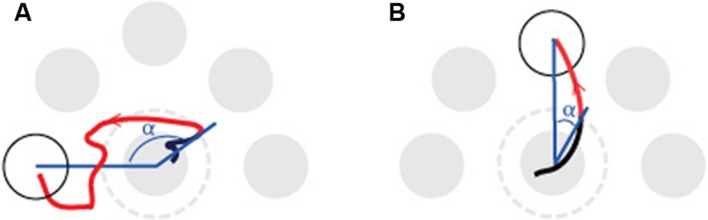
**Illustration of the center-out movement to a presented radial target (white) and the outcome parameters directional error and homing time.** The figure is based on one trial of a young subject at the start **(A)** and at the end **(B)** of a practice session. The angle (α) between the two blue lines determines the directional error. The homing time is the time it takes from leaving the dotted circle to stabilizing on the radial target (red trajectory). At the start of the experiment the subject had a larger homing time and a larger directional error than at the end of the practice session (left panel vs. right panel).

Before determining the outcome measures from the CoP coordinates the raw CoP position data were filtered using a low-pass fourth order Butterworth filter, with a cut-off frequency of 5 Hz. Subsequently, plan-based and on line control processes were isolated with a trajectory analysis of each target-directed movement (see **Figure 2**). Specifically, the directional error of the first part of the CoP trajectory and the time it takes to reach and stabilize on the radial target in the second part (homing-time) of the CoP trajectory were selected to reflect the plan-based and on-line control processes, respectively. The directional error was determined at the point where the cursor leaves the central target area (e.g., the exit point) and defined as the angle between two lines; one line connecting the exit point with the origin of the central target and the other representing the ideal path connecting the origins of the central target and the radial target. The homing time is the time it takes from the exit point until the disappearance of the radial target.

The transition from the sequence posttest (block S15; S-Post) to the random posttest (R3; R-Post) resulted in large movement errors on the first trial of the random block. This error was possibly the result of learning the sequence, causing participants to wrongly predict an upcoming target location in the random target location condition. Therefore, the first trial of the 21 trials of the random posttest (block R3) has been removed from analysis. We excluded from further analysis also the trials in which the cursor traveled a distance less than 0.01 m in the home area (6.14% due to technical difficulties) and trials in which movement times were longer than 2.3 s (0.86%).

### Statistical Analysis

All statistical analyses were applied to both outcome measures, homing time and directional error, separately. Preliminary 2 (Age) × 3 (Condition) repeated-measures ANOVAs with Age as the between-subjects factor and Condition as the within-subjects factor were applied to the performance measures in the random baseline-test (R-Base) to compare the initial task performance of the young and older participants for the three conditions. To assess the general training effects over the sequence blocks, Age (young vs. old) × Condition (implicit vs. explicit single vs. explicit dual task) × Test block (R-Pre vs. S-post) ANOVAs were applied. To examine whether sequence-specific learning occurred, sequence removal and sequence reintroduction effects were tested with Test block (S-Post vs. R-Post vs. S-Rec) × Condition (explicit single task vs. explicit dual-task vs. implicit) × Age (young vs. old) ANOVAs. To further explore significant effects, we performed *post hoc* tests with Bonferroni corrected adjustments to protect the level of significance.

## Results

### Explicit Knowledge Assessment

In the explicit single-task condition participants were instructed to discover the sequence while performing the target-directed weight-shifting task. Upon completion, one older participant did not show explicit knowledge of the sequence in both the free-recall and the forced choice task, thus his results were excluded from further analysis. All young participants acquired explicit sequence knowledge in the explicit single-task condition and successfully reported the sequence order in the free recall test. In the implicit condition, where sequence knowledge should not be available to the participants, two young and one older participant revealed the right sequence in the explicit knowledge assessment tests. The three participants that discovered the sequence order in the implicit condition were excluded from further analysis. After performing the explicit-dual task condition all the young participants and none of the older participants were able to recall the sequence. When the older adults without declarative knowledge of the sequence were given the forced-choice test, four of them were belatedly able to recognize the sequence order.

Interestingly, not all participants that discovered the sequence order in the training phase also noticed the recurrence of the sequence in block S16 (test block: S-rec), because it was hidden between two random blocks (R-Post and R4). Only six young and five older participants reported that they noticed the recurrence of the sequence in the explicit single-task condition. Three young and two older participants mentioned the sequence recurrence in the explicit dual-task condition and none of the participants noticed the sequence recurrence in the implicit learning condition.

### Cognitive Task Performance

During the experiment participants were asked to prioritize the visuo-spatial memory task over sequence learning. Task performance on the visuo-spatial memory test during sequence learning was compared with task performance prior to the experiment. As expected, no significant decrease in performance on the cognitive task was observed for both the young and older participants. When tested prior to the experiment the young participants performed the visuo-spatial memory task successfully, that is 17 out of 17 numbers were correctly placed. Young participants correctly placed an average of 15.8 out of 17 numbers at the end of the dual task conditions (explicit dual task and implicit condition). The older participants scored an average of 6.9 out of 13 correct numbers before the intervention, while this was 6.3 out of 13 at the end of the dual task conditions. These results imply that participants followed the instructions and were able to focus on the cognitive-task while performing the target-directed weight-shifting task.

### Baseline Motor Task Performance

**FIGURE 3:**
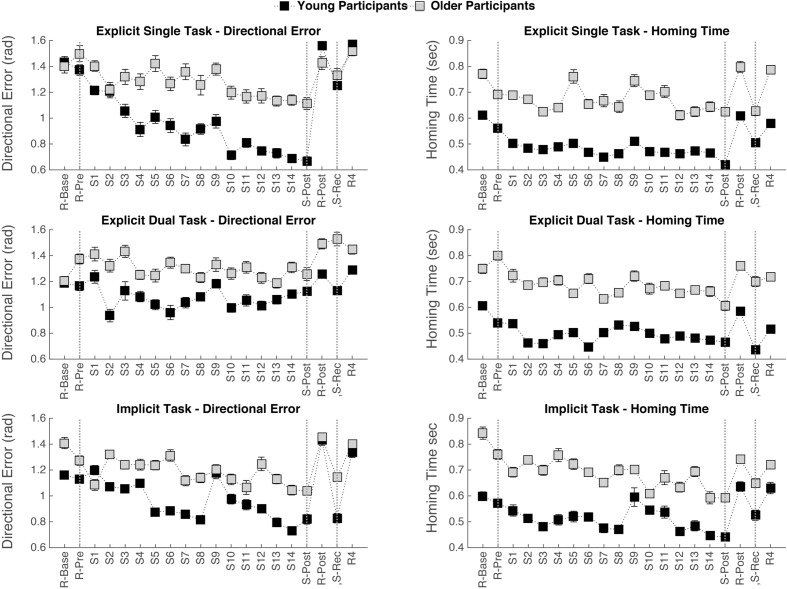
**Mean value and standard-error bar for each age group on each test block showing; the absence of an aging effect at the beginning of the experiment for directional error, a slower homing-time for the older participants, general practice effects on directional error and homing-time (R-Pre vs. S-Post), and the effects of sequence removal and re-introduction (the phase between the two vertical dotted lines on the right side)**.

Mean and standard deviations of homing-time and directional error for old and young participants per condition on the first block of trials with random target order, e.g., R-Base are depicted in **Figure 3**. A significant main effect of Age was found for homing-time [*F*(1,18) = 10.22, *p* = 0.005], with a longer homing-time for the old than the young participants. No significant main effect of Age was found for directional error. No significant main effects of Condition nor significant interaction effects of Age by Condition were revealed, indicating no significant performance difference between the various conditions at the start of the experiment.

### Practice Phase (R-Pre vs. S-Post)

Mean and standard deviations of homing-time and directional error for old and young participants per condition on the test moments R-Pre and S-post are presented in **Figure 3**.

For homing-time, significant main effects of Age [*F*(1,18) = 17.55, *p* = 0.001] and Test block [*F*(1,18) = 34.25, *p* < 0.001] were found. Homing-time was shorter for the young than for the old participants and performance improved with practice in all learning conditions, indicated by a decrease in homing-time. No significant main effect of Condition and no interaction effects were found.

For directional error, a significant main effect of Test block [*F*(1,18) = 14.40, *p* = 0.001] and a significant interaction effect of Test block by Condition [*F*(2,36) = 5.34, *p* = 0.009] was found. No significant main effect of Age was found. To assess the difference between R-pre and S-post for each learning condition separately, *post hoc* paired *t*-tests were performed with an adjusted alpha of 0.017 to protect the level of significance. Performance improvement over practice was revealed, indicated by a significant decrease in directional error, in the explicit single task condition [*t*(19) = 4.13, *p* = 0.001] and in the implicit condition [*t*(19) = 2.85, *p* = 0.010], but not in the explicit dual task condition.

### Sequence Removal and Sequence Recurrence Analysis (S-Post vs. R-Post vs. S-Rec)

Means and standard deviations of homing-time and directional error for old and young participants per condition on the test moments S-Post, R-Post, and S-Rec are presented in **Figure 3**.

For homing-time, a significant main effect of Age [*F*(1,18) = 16.23, *p* = 0.001] and a significant main effect of Test block (S-Post, R-Post, S-Rec) was observed [*F*(2,36) = 34.36, *p* < 0.001]. No significant main effect of Condition and no significant interaction effects were found. *Post hoc* pairwise comparisons with Bonferroni adjustments revealed that homing-time significantly increased in R-Post compared to S-Post (*p* < 0.001) and then significantly decreased in S-Rec compared to R-Post (*p* < 0.001). The homing time was not significantly different between the test blocks S-Post and S-Rec (*p* = 0.072). This implies that both young and older adults were able to improve the time it takes to home in on the target by taking advantage of the sequence structure, as indicated by a significant increase in homing-time when the sequence is removed and a decrease again when the sequence is reintroduced.

For directional error, significant main effects of Test block (S-Post, R-Post, S-Rec); [*F*(2,36) = 28.29, *p* < 0.001) and Condition [*F*(2,36) = 3.51, *p* = 0.040] were found. Furthermore, there was a significant interaction between Test block and Condition [*F*(4,72) = 6.80, *p* < 0.001] and also a significant interaction between Age, Test block, and Condition [*F*(4,72) = 3.09, *p* = 0.021]. To assess the difference between S-post and R-post for each learning condition and age group separately, *post hoc* pairwise comparisons were performed with a Bonferroni adjusted alpha of 0.008 to protect the level of significance. For the older participants a significantly higher directional error with sequence removal was only observed in the implicit condition and not in both explicit conditions. For the young participants a significantly higher directional error with sequence removal was observed in the implicit and explicit single task conditions but not in the explicit dual task condition. To further explore the robustness of the sequence learning effect, differences between the two sequence blocks (S-post and S-rec) were assessed. In the implicit condition the difference in directional error between S-post and S-rec was not significant for both young and older adults, indicating a robust sequence learning effect. Only for the young adults in the explicit single task condition a significant increase in directional error was observed in S-rec compared to S-post (*p* < 0.001), suggesting an interfering effect of the random block (R-post), which was inserted between the two sequence blocks (S-post and S-rec).

## Discussion

The aim of the current study was to examine the possible effect(s) of implicit and explicit learning of a sequential postural task in older adults compared to young adults. To this end, a target-directed weight-shifting task was created in a virtual environment in which targets were presented in a certain sequence or randomly.

The results showed that participants were able to discover the simple deterministic sequence order of five targets in the explicit learning condition. In the implicit condition the concurrent cognitive task prevented most participants from discovering the simple sequence, as intended. In accordance with our hypothesis, and in contrast to previous research using a postural perturbation task (Van Ooteghem et al., 2008, 2010), we found evidence for sequence-specific postural motor learning. With practice on the sequence blocks young and older participants showed improved performance and an abrupt decrease in performance when the sequential regularity was removed. However, depending on age plan-based motor sequence learning, quantified by improvements in directional error, was not optimal in all learning conditions. Conversely sequence specific improvements in homing time occurred in all participants regardless of learning condition and age. The general observation that old and young participants show sequence specific motor learning in a postural control task is in contrast with the work of Van Ooteghem et al. (2008, 2010). In a postural motor learning study in which participants were exposed to a repeated sequence of platform motions, sequence learning did not occur. Participants improved with practice, but learning was not better for the repeating sequence than for the random sequence (Van Ooteghem et al., 2008, 2010). It should be noted that in our study the task setup was different, instead of reacting to external perturbations, participants generated active postural responses to aim for visually presented targets in a sequential order and showed sequence specific postural motor learning.

Adding explicit knowledge of the sequence order was not a prerequisite for acquiring sequence-specific improvements in target-directed weight-shifting. In both age groups in the implicit condition, where explicit knowledge of the sequence order was not acquired, homing time and directional error improved during prolonged sequence practice. Subsequently, after practice, homing time and directional error increased with sequence removal (in test block R-post) and decreased again with sequence reintroduction (in test block S-rec). This implies that without knowledge of the sequence order and when distracted by a cognitive task (in the implicit condition), both older and young participants showed sequence-specific improvements in the steadiness of the movement execution and in plan-based control.

For improving plan-based control (directional error), explicit information about the sequence order through self-discovery, was in certain circumstances even detrimental. Participants who intended to search for the sequence order and concurrently performed the visuo-spatial cognitive task, did not improve in directional error. Thus, our hypothesis that a concurrent cognitive task disrupts postural sequence learning with less interference on implicit than explicit motor learning was supported for learning to control the direction of the initial part of the target-directed movement. Studies using the serial reaction time task found similar results; explicitly searching for a sequence structure could disrupt motor sequence learning in conditions in which there is not sufficient cognitive capacity available (Curran, 1997; Fletcher et al., 2005; Jimenez and Vazquez, 2005). The multiple explicit requirements of the explicit dual task condition is likely to have placed a high load on the processing limitations of the working memory system, thereby retarding improvements in the advance planning of the upcoming target-directed postural movement in both young and older participants.

Sequence-specific improvements in the homing time occurred in both older and young participants regardless of the learning condition. Thus motor learning in the homing phase was preserved when the cognitive capacity was overloaded by the explicit attempts to acquire the sequence order in combination with performing the visuo-spatial cognitive task. Therefore, it can be assumed that changes in the homing phase are implicitly achieved, unmolested by acquiring explicit sequence knowledge and without much dependence on working memory (Willingham and Goedert-Eschmann, 1999; Willingham et al., 2002).

Age-related difficulties with sequential motor learning were observed for plan-based control (directional error) in the explicit single task condition. Both young and older participants acquired knowledge of the sequence order in this learning condition, but only the young participants and not the older participants showed sequence-specific improvements in directional error, as revealed by the significant increase in directional error with sequence removal (in test block R-post). However, for the young participants, this sequence specific learning was less robust compared to the implicit condition. This was evidenced by the fact that reintroducing the sequence unbeknownst to participants (in test block S-Rec) did not significantly decrease directional error. Even though explicit knowledge was acquired, one did not recover from the increase in directional error due to the random block interference (in test block R-Post). Possibly one needs to be fully aware of the recurrence of the sequence before being able to express explicit sequence learning in plan-based control of the movement (Willingham and Goedert-Eschmann, 1999). In contrast, in the implicit condition plan-based control improved (decrease in directional error) when the sequence was reintroduced (in test block S-Rec). This indicates a robust implicit memory of the sequence and the ability to more accurately execute the initial part of the target-directed movement without sequence awareness. The finding that, for the older participants, sequence specific learning of directional error occurred in the implicit condition and not in both explicit conditions, supports the proposition that implicit motor learning is relatively preserved with age in comparison with explicit learning (Reber, 1992; Cherry and Stadler, 1995; Hedden and Gabrieli, 2004; Shea et al., 2006; Brown et al., 2009; Gaillard et al., 2009; Song et al., 2009).

A new finding of our work is that sequence practice not only affects the initial plan-based part of the movement, but also led to a progressive improvement of motor control late in the movement trajectory in both young and older adults. Successful achievement of the task goal depends not only on correctly anticipating the target and planning a movement with the right direction and extent, but also on the ability to quickly adjust the ongoing movement based on visual feedback about the effector and target (Glover, 2004; Caljouw et al., 2006, 2011). When sequential arm-reaching movements are practiced, control shifts from a reaction mode to an anticipatory mode within a few practice trials, requiring far less online visual control at the end of the practice session than at the start of the practice session (Ghilardi et al., 2003, 2009). In our target-directed weight-shifting task, the target area is not easily reached given the inherent variability in postural control, which is observed even during quiet standing in the start position (Zatsiorsky and Duarte, 2000; Lamoth et al., 2009). Even after practice, a directional error with a large standard deviation was still present. The presence of a directional error highlights the need for corrections in the vicinity of the target area, to achieve a rather stable final position within the target. The homing time in older participants was substantially longer than young participants, indicating less optimal control in the vicinity of the target (Jongman et al., 2012; de Vries et al., 2014). Despite this difference in performance, both older and younger participants showed changes in homing time with sequence removal and reintroduction after practice, indicating age-invariant sequence-specific performance optimization late in the movement trajectory.

Participants showed a progressive improvement in aim direction and homing time for the sequence elements without explicit awareness of the sequence in the implicit condition. This is inconsistent with previous work on upper-limb motor sequence learning suggesting that sequence awareness allows for a progressive change in movement execution (Moisello et al., 2009, 2011; Oostwoud Wijdenes et al., 2016). For example, in a finger-opposition task procedural optimization of the movement (reflected by a change in thumb-finger touch duration) was reached only in conditions in which participants acquired explicit sequence knowledge (Moisello et al., 2011). The observations in our study do not support the suggestion that knowledge is important for motor learning (Stanley and Krakauer, 2013; Wong et al., 2015).

## Conclusion

The results of the present study show that sequence learning in a postural visuomotor control task is possible in both young and older adults. The most robust learning effects for both groups were observed for the implicit learning condition. Only the young participants were able to decrease the directional error with sequence practice in the explicit condition, implying that older adults were hampered by the additional attentional cost of explicit sequence monitoring. In contrast, focusing attention on task-irrelevant aspects during sequence practice (i.e., the cognitive task in the implicit condition) did not hamper improvement in both aim direction and homing time in older adults. The finding that introducing a secondary task that prevented the accumulation of explicit knowledge resulted in a robust learning effect provides further support for the notion that implicit motor learning methods may be desirable for older adults.

## Author Contributions

SC and CL made substantial contributions to conception and design, acquisition of data, and analysis and interpretation of data. RV made substantial contribution to analysis and interpretation of data. SC, CL, and RV participated in drafting the article and revising it critically for important intellectual content; and give final approval of the version to be submitted and are accountable for all aspects of the work in ensuring that questions related to the accuracy or integrity of any part of the work are appropriately investigated and resolved.

## Conflict of Interest Statement

The authors declare that the research was conducted in the absence of any commercial or financial relationships that could be construed as a potential conflict of interest.
